# Overexpression of *AtNCED3* gene improved drought tolerance in soybean in greenhouse and field conditions

**DOI:** 10.1590/1678-4685-GMB-2019-0292

**Published:** 2020-06-08

**Authors:** Mayla Daiane Correa Molinari, Renata Fuganti-Pagliarini, Silvana Regina Rockenbach Marin, Leonardo Cesar Ferreira, Daniel de Amorim Barbosa, Juliana Marcolino-Gomes, Maria Cristina Neves de Oliveira, Liliane Marcia Mertz-Henning, Norihito Kanamori, Hironori Takasaki, Kaoru Urano, Kazuo Shinozaki, Kazuo Nakashima, Kazuko Yamaguchi-Shinozaki, Alexandre Lima Nepomuceno

**Affiliations:** 1 Universidade Estadual de Londrina, Departamento Geral de Biologia, Londrina, PR, Brazil.; 2 Embrapa Soja, Londrina, PR, Brazil.; 3 Japan International Research Center for Agricultural Sciences, Biological Resources and Post-harvest Division, Tsukuba, Ibaraki, Japan.; 4 RIKEN Center for Sustainable Resource Science, Gene Discovery Research Group, Tsukuba, Ibaraki, Japan.; 5 The University of Tokyo, Laboratory of Plant Molecular Physiology, Department of Applied Biological Chemistry, Bunkyo-ku, Tokyo, Japan.

**Keywords:** *Glycine max* L. Merrill, water deficit, abscisic acid, gas exchange measurements, RT-qPCR

## Abstract

Water deficit is an important climatic problem that can impair agriculture yield and economy. Genetically modified soybean plants containing the *AtNCED3* gene were obtained aiming drought-tolerance improvement. The *NCED3* gene encodes a 9-cis-epoxycarotenoid dioxygenase (NCED, EC 1.13.11.51), an important enzyme in abscisic acid biosynthesis. ABA activates the expression of drought-responsive genes, in water-deficit conditions, targeting defense mechanisms and enabling plants to survive under low water availability. Results from greenhouse experiments showed that the transgene *AtNCED3* and the endogenous genes *GmAREB1*, *GmPP2C*, *GmSnRK2* and *GmAAO3* presented higher expression under water deficit (WD) in the event 2Ha11 than in WT-plants. No significant correlation was observed between the plant materials and WD conditions for growth parameters; however, gas exchange measurements decreased in the GM event, which also showed 80% higher intrinsic water use when compared to WT plants. In crop season 2015/16, event 2Ha11 showed higher total number of pods, higher number of pods with seeds and yield than WT plants. ABA concentration was also higher in GM plants under WD. These results obtained in field screenings suggest that *AtNCED3* soybean plants might outperform under drought, reducing economic and yield losses, thus being a good candidate line to be incorporated in the soybean-breeding program to develop drought-tolerant cultivars.

## Introduction

Brazil is the second country in the worldwide production and processing of soybeans, and the second largest exporter of grains and oil (Aprosoja, 2016). This productive chain generates 1.4 million jobs, consisting of the main contributor to the Brazilian Gross Domestic Product (GDP) (IBGE, 2016). According to IBGE (Brazilian Institute for Geography and Statistics), in 2015, agriculture was the only economic sector that did not reduce its contribution to GDP, increasing 1.8% in relation to the previous year, on the influence of soybeans and corn (FAO, 2016).

Despite the positive numbers, the increased frequency and intensity of drought periods have generated recurrent and significant losses in soybean yield, indicating that sustainable crop production is highly dependent on the development of cultivars more tolerant to water deficit (WD), which can be obtained through available genetic engineering techniques. Many genes conferring drought tolerance had been identified under the current global climate change scenario (Ramiro *et al.*, 2016), and many of them were introduced in important crops. However, sometimes the proof of drought tolerance improvement in real field conditions is not achieved, keeping the GM events characterization only in greenhouse.

Plants have two main regulatory pathways for drought responses: an ABA-dependent and an ABA-independent pathway (Shinozaki and Yamaguchi-Shinozaki, 2007). Besides controlling several plant development processes, the ABA hormone coordinates an intricate regulatory network that enables plants to survive under short-water availability conditions (Kim *et al.*, 2010). Under WD, ABA levels increase in the plant system, which leads ABA to bind to specific receptors (PYLs) forming a complex with other enzymes (PP2Cs and SnRKs). This binding of ABA releases kinases that bind to specific transcription factors (TFs) such as ABRE/ABFs, which target drought-responsive genes expression, activating defense mechanisms (Barbosa *et al.*, 2013; Cao *et al.*, 2013; Takeuchi *et al.*, 2014; Park *et al.*, 2015), such as stomatal closure, and synthesis of osmoprotectants enzymes (Sah *et al.*, 2016).

Several studies have reported (Iuchi *et al.*, 2001; Endo *et al.*, 2008), a better performance of plants under WD overexpressing genes that encode enzymes of the ABA bio-synthetic pathway, in particular the *NCED3* gene, which encodes 9-cis-epoxycarotenoid dioxygenase (NCED, EC 1.13.11.51), a central enzyme in ABA biosynthesis (Bhaskara *et al.*, 2012; Behnam *et al.*, 2013). The over-expression of *AtNCED3*, in *Arabidopsis*, increased endogenous ABA levels, targeted the transcription of drought- and ABA-inducible genes, reduced transpiration rate, and improved drought tolerance in the GM plants (Iuchi *et al.*, 2001). The *NCED3* gene has also been described to be strongly induced in WD, under greenhouse conditions, in several economically important crops such as avocado (*Persea americana*) (Chernys and Zeevaart, 2000), citrus (*Citrus sinensis*) (Rodrigo *et al.*, 2006; Neves *et al.*, 2013; Pedrosa *et al.*, 2015), common bean (*Phaseolus vulgaris*) (Qin and Zeevaart, 1999), cowpea (*Vigna unguiculata*) (Iuchi *et al.*, 2000), peanuts (*Arachis hypogaea*) (Wan and Li, 2006), tomato (*Solanum lycopersicum*) (Burbidge *et al.*, 1999), and turmeric (*Curcuma longa*) (Ahrazem *et al.*, 2012).

In soybean, no information regarding GM plants with the *NCED* gene is available. Thus, considering the eminent need for plants more tolerant to WD periods in the future scenario of world climate changes, our main objective was to develop GM soybeans with improved drought tolerance by introducing the *AtNCED3* gene into a conventional soybean cultivar. Under greenhouse conditions, these plants were submitted to WD, and ABA content was quantified in leaves. In addition, the GM plants were submitted to WD induced under real field conditions for two crop seasons. As in greenhouse environment, growing conditions can be monitored; plants behavior may not reproduce accurately the way plants outperform water deficit over a complete growing-season in a real field spot (Passioura, 2012). Therefore, it's important to challenge the applied strategy to cope with drought, in water-sensitive phases such as flowering - pod-filling, when plants require 7–8 mm of water daily; and water scarcity implies in significant production losses (Berlato *et al.*, 1986; Embrapa, 2015). Moreover, as in other countries, prior to approving a commercial product, regulatory bodies such as the Brazilian National Technical Biosafety Commission requests tests in field conditions. Thus, with these lines in field can speed up the deregulamentation process.

Therefore, our work brings new insights into how the overexpression of a gene encoding NCED enzyme from ABA biosynthesis acts in WD tolerance in soybean plants, and how these GM lines perform in field conditions, where and when data can precisely gauge whether the technology is efficacious. These data might help soybean breeders to indicate the best performing lines in real crop circumstances to introduce them into breeding programs aiming to develop a cultivar to be used and marketed to soy producers.

## Material and Methods

### Biological material and identification of GM plants positive to 35S: AtNCED3

Vectors pMDC123-GI and pC3300J-35S (Cambia Enabling Innovation) were used as backbone to construct the expression cassette contained the *AtNCED3* gene. This cassette was introduced, via electroporation; into the *A. tumefaciens* strain EHA 105 (Hood *et al.*, 1993) as described by Casali and Preston (2003). This vector is under the control of the constitutive promoter CaMV 35S (Cauliflower mosaic virus) and TNOS terminator (*A. tumefaciens* nopaline synthase). The marker genes present in the cassette are *bar* gene (phosphinothricin acetyl transferase), used as a selective agent once it confers resistance to ammonium glufosinate herbicide; and *NPTII* gene (Neomycin phosphotransferase), which confers resistance to the antibiotic kanamycin, and was applied to select colonies carrying the inserted transgene (Figure S1).

The BRS 184 (wild type (WT) - genetic background) conventional soybean cultivar was transformed using the *Agrobacterium tumefaciens* method described by Paz *et al.* (2006). Aiming at improving injury for infection, a protocol modification was introduced; using a stainless-steel micro brush, each cotyledon was scratched 10 to 12 times.

During the selection process, the seedlings that developed well were transferred to a mixture of substrate/sand (1:1), with the substrate comprising soil/sand/organic compounds at 3:2:2 parts. The seedlings were then acclimated for at least 1 week in a growing chamber and subsequently transferred to a greenhouse, where molecular evaluations were performed.

A conventional PCR using a set of primers A (Table S1A) was carried out to confirm positive plants. The genomic DNA was extracted from leaf tissues, according to Doyle and Doyle, 1987. PCR reactions were carried out in a final volume of 25 μL and were composed of 5 μM of each forward and reverse primer, 0.4 mM dNTPs, 2 mM magnesium chloride, 1U *Taq* DNA polymerase, and 50 ng μL^-1^ DNA. Amplifications were performed in a Veritti^®^ (Applied Biosystems) thermocycler. The cycling parameters used were: an initial denaturation at 95 °C for 5 min, followed by 35 cycles of 95 °C for 30 s, 55 °C for 30 s, and 72 °C for 30 s, with a final elongation cycle of 72 °C for 5 min. The PCR products were evaluated in a 1% agarose gel electrophoresis (1x SB) stained with ethidium bromide. The plants identified as positive were used in experiments for molecular, physiological and agronomical evaluations.

### Segregation pattern analysis and copy number quantification of the transgene via qPCR

The segregation pattern in the generated events was analyzed through conventional PCR, in plants from T1 and T2 generations. The X^2^ test (*p*≤0.05) was applied to check if *AtNCED3* gene was segregating in agreement with the expected Mendelian pattern.

Transgene-inserted copy number quantification was achieved using *AtNCED3* as the target gene and *Lec* gene (*GmLec,* Accession No. K00821), as endogenous and calibrator. This is a soybean species-specific gene that presents only one copy in the haploid genome (Meyer *et al.*, 1994), or two allelic copies (homozygous).

An amplification efficiency curve was performed, for both target and calibrator genes, using a series of DNA dilutions (5^–6^, 5^–5^, 5^–4^, 5^–3^ and 5^–2^). The amplification reactions were composed of DNA dilutions (one reaction for each dilution), 0.2 μM F and R primers (Table S1B), and 1x reaction buffer Platinum^®^ SYBR Green^®^ qPCR SuperMix UDG (Invitrogen) composed of *Taq* DNA polymerase, Tris-HCl, KCl, 6 mM MgCl_2_, 400 μM dGTP, 400 μM dATP, 400 μM dCTP, 800 μM dUTP, uracil DNA glycosylase (UDG), and stabilizers. Reactions were carried out in three biological and technical replicates in a 7900HT (Applied Biosystems) thermocycler. The cycling parameters used were: 50 °C for 2 min, denaturation at 95 °C for 10 min, followed by 40 cycles of 95 °C for 15 s, 60 °C for 1 min, 95 °C for 15 s, 60 °C for 15 s, and 95 °C for 15 s.

The PCR reaction efficiency was calculated using the E = [5-1/slope] −1 formula (Pfaffl, 2001). *AtNCED3* gene inserted copy number was quantified by subtracting Ct values of the target gene from the Ct value of the endogenous reference gene (*GmLec*, Accession No. K00821), resulting in the ΔCt value for each sample. The copy number was calculated as 2 elevated to the average of -ΔCt (^2-ΔCt^), where 2 corresponds to the sum of the target gene (100% = 1) and the endogenous control (100% = 1) efficiencies, and it differs depending on primer efficiency in the amplification reaction (Livak and Schmittgen, 2001).

### Analysis of gene expression by RT-qPCR

The total RNA was extracted from soybean leaf samples using Trizol^®^ reagent. After that, samples were treated with DNase I kit (Invitrogen, Carlsbad, CA) to remove possible remaining DNA and lastly the cDNA was synthesized using SuperScript^®^ III First-Strand Synthesis System (Invitrogen/ Catalog number: 180800) according to the manufacturer's instructions. The relative gene expression quantification was carried out in three biological and technical replicates (n = 9). Reactions were composed of cDNAs, 0.2 μM F and R primers and 1x reaction buffer Platinum^®^ SYBR Green^®^ qPCR SuperMix UDG (Invitrogen). The reactions were conducted in a 7900HT thermocycler (Applied Biosystems), following the same cycling conditions described above.

The sequences used to design primers for RT-qPCR were obtained from Phytozome. The CDS sequence from the *AtNCED3* gene from plasmid was aligned with soybean endogenous genes *GmNCED3* (Glyma.05G140900.1 and Glyma.08G096200.1) using CLUSTWAL W. A set of primers was designed to amplify a genomic region specific from *Arabidopsis thaliana*, ensuring that any differences in gene expression and copy number quantification did not result from the expression of the endogenous gene *GmNCED3*.

The expression level of ABA-dependent genes, such as *GmAREB1* (Glyma.04G039300; Glyma.07G213100; Glyma.02G131700), *GmPP2C* (Glyma.14G195200), *GmSnRK2* (Glyma.02G135500), and *GmAAO3* (Glyma.14gG045100) was quantified. The *GmAREB1* genes were aligned and primers designed in a conserved region. Specific primers for the endogenous genes *GmPP2C*, *GmSnRK2* and *GmAAO3* were also designed using the Primer3Plus software (Table S1C).

The gene expression relative quantification was carried out in three biological and technical replicates (n = 9). The reactions were comprised of cDNAs, 0.2 μM F and R primers and 1x reaction buffer Platinum^®^ SYBR Green^®^ qPCR SuperMix UDG (Invitrogen). Reactions were conducted in a 7900HT thermocycler (Applied Biosystems), following the same cycling conditions described above. The gene expression level was determined using the formula 2^-ΔΔct^ (Rodrigues *et al.*, 2015). Statistical analysis was carried out by applying the *t*-test (*p*≤0.05).

Considering segregation and transgene quantification results, the event *AtNCED3* 2Ha11 was chosen to be evaluated regarding growth, physiological and agronomical traits in experiments carried out under greenhouse and field conditions.

### Evaluation of GM event submitted to WD under greenhouse conditions

T2 generation seeds from *AtNCED3* 2Ha11 event and BRS 184 soybean conventional cultivar (WT plants) were treated with Vitavax^®^ Thiram 200 SC (200 g L^-1^) (ADAPAR) for health quality purposes. After that, the seeds were germinated on Germitest^®^ paper, for 96 h at 25 ± 1 °C and 100% relative humidity (RH). Then, seedlings were relocated to 1l pots filled with substrate mixture (soil-sand-organic compound 3:2:2), each pot containing one seedling. The pots with seedlings were maintained in a greenhouse at 28 ± 2 °C, with records of temperature and RH (relative humidity) every 5 min through a thermohygrograph (Hobo U14-002, Onset^®^). Experimental design was in completely randomized blocks, in a 2x2 factorial arrangement, i.e. two plant materials (2Ha11 and BRS 184) and two water conditions (control – C and water deficit – WD), with nine blocks.

A conventional PCR using primers specific for the transgene was carried out when plants reached V1 developmental stage (Fehr *et al.*, 1971). The plants were kept at 100% field capacity (FC) until phenological stage V4, by irrigating them with a fixed volume of water sufficient to saturate the substrate. At V4 stage, one day before WD treatment, at the end of the afternoon, all pots were saturated with water and excess water drained overnight. In the following morning, the pots were giftwrapped in polyethylene bags and the central region of each pot was covered with cotton around the stem base to prevent water loss by evaporation. Control plants group were maintained at 100% FC, while in the WD group irrigation was withheld. Stomatal conductance (*gs*) was monitored daily. When WD-group plants presented *gs* values less than 200 mmol H_2_O m^-2^ s^-1^ (Flexas *et al.*, 2004; Salinet, 2009) which occurred eight days after withholding irrigation, gas exchange parameters – photosynthetic rate (*A*), sub-stomatal CO_2_ (*Ci*), transpiration rate (*E*), and *gs* – were measured, using a portable infrared gas analyzer (LCpro-SD, ADC BioScientific). The measurements were carried out inside the greenhouse from 9.00 am (Brazilian daylight-saving time) at 1000 mmol m^-2^ s^-1^ photo-synthetically active radiation (PAR), on the central leaflet of the third fully-expanded trifoliate leaf (apex-base direction) in three different plants. The intrinsic water use efficiency (WUE) was achieved through the ratio *A/gs*. Afterward, the same trifoliate leaf was collected, wrapped in aluminum foil, immersed in liquid nitrogen and stored at −80 °C. These biological materials were used to perform gene expression analysis, by RT-qPCR.

Seven days after withholding irrigation, the number of nodes (NN) and the total leaf area were assayed, using a leaf area meter (LI-3100C model, Licor). Leaf blades, stems, petioles and roots were dried to constant weight, in a forced aeration oven at 60 °C, to weight shoot dry matter (leaf blades + stems + petioles) and root dry matter (per plant). The plant height was measured in two points: at the start (H1) and at the end (H2) of WD-treatment period. The mean length of internodes corresponded to the ratio between H2 and the number of nodes. The relative growth rate in height (RGRH) was estimated using RGRH (%) = [((H2 – H1)/H1) ×100] equation.

After the evaluation of growth parameters and sampling for gene expression analysis, the plants were moved to 8l pots filled with a substrate composed of soil-sand-organic compound (3:2:2). All pots were maintained under continuous irrigation until the end of the cycle, when agronomical traits (per plant) such as number of seeds, number of pods presenting seeds, total number of seeds, and yield were evaluated.

### Evaluation of GM events submitted to WD-treatment in field conditions

The experiment was conducted during the crop seasons 2015/16 and 2016/2017, in a field area located at National Soybean Research Center (63°11'S, 51°11'W, 630 m altitude) a Brazilian Agricultural Research Corporation (Embrapa Soybean, Londrina, PR, Brazil) unit. All required documentation to test the GM lines in field conditions was submitted to and approved by CTNBio (Process n° 01200.002859/2015-54 published in the Brazilian Official Journal on August 10, 2015, Technical advice #4.648/2015).

A randomized complete block split-plot design was applied, with four blocks. The plots corresponded to two water treatments: irrigated (IRR: water from rainfall + irrigation applied when soil matric potential reached values between −0.03 and −0.05 MPa) and non-irrigated (NIRR: water from rainfall). The subplots corresponded to the BRS 184 soybean conventional cultivar and its isoline, the transgenic event *AtNCED3* 2Ha11. Each subplot corresponded to 220 m^2^.

The cultivation practices during the experiment followed procedures routinely approved at Embrapa Soja (Embrapa, 2011). BRS 360RR cultivar soybean plants were used as a 10 m isolation border, according to the Brazilian legislation. RH and air temperature were daily monitored through a weather station located near to the experiment area. Plants were evaluated for number and dry matter of seeds, pods with seeds, 100-seed weight and yield at the end of the cycle. The sequential water balance was assayed according to Thornthwaite and Mather (1955).

### ABA quantification

For the ABA quantification, an experiment was performed in greenhouse conditions following the same procedures previously described. However, WD imposition was maintained for 10 days. Thus, leaf samples (1 g) from the 2Ha11 GM line and BRS 184 soybean conventional cultivar were collected, submerged in liquid nitrogen and ware-housed at −80 °C. An aliquot (1 mg) of leaf tissue was powdered in a 1.5 ml microtube containing metal beads and 500 μL extraction buffer was added (80% methanol, 0.5 g L^-1^ monohydrate citric acid, 100 mg L^-1^ butyl hydroxytoluene). The solution was maintained overnight, at −4 °C. The next day, the solution was centrifuged for 10 min, at 4 °C, at 6,000 *g* and the pellet resuspended in 500 μL TBS buffer (45 mm Tris-HCl, pH 7.8; 90 μM MgCl_2_, 0.135 M NaCl, 10% methanol). The ABA concentration was quantified using Phytodetek immunoassay kit (ABA Agdia Inc.), following to the manufacturer's instructions.

### Statistical analysis

Data displayed normal distribution and were submitted to analysis of variance (ANOVA) and comparison of means by Tukey test (*p*≤0.05). The gas exchange parameters were compared between plant materials (BRS 184 soybean conventional cultivar and 2Ha11GM line) and between water treatments (C- control and WD-water deficit). Plant height, leaf area, number of seeds per plant, 100-seed weight, yield and total number of pods were compared between the plant materials (BRS 184 soybean conventional cultivar and 2Ha11GM line) and between crop seasons (2015/2016 and 2016/2017).

## Results

### Identification of GM plants positive to 35S: AtNCED3, segregation pattern analysis and copy number quantification of the transgene via qPCR

One hundred and eighty-four cotyledons from BRS 184 soybean conventional cultivar were transformed with the construct *35S: AtNCED3,* through the *Agrobacterium tumefaciens* method. Two positive events were identified in T0 generation and named 2Ha11 and 2Ha13, with a transformation efficiency of 1.08%. This efficiency was calculated by dividing the number of positive plants per total number of explants transformed × 100. In T1 generation, seven plants were identified as positive - five from the event 2Ha11 and two from the event 2Ha13.

The segregation pattern analysis and the copy number quantification of the transgene via qPCR were carried out in plants from T2 generation. The event 2Ha11 followed the Mendelian segregation pattern, but the event 2Ha13 did not (Table 1). Although a second GM line was obtained and initially characterized, the transgene in line 2Ha13 was an instable insertion, not being transferred to further generations than F_1_; thus, it was discarded from further analysis.

**Table 1:** Chi-square (X^2^) test for Mendelian segregation pattern, in T_2_ generation, for *35S: AtNCED3* construction.

Events T_1_	Total/seeds	Positive plants	Negative plants	X^2^	Segregation (3:1)
2Ha 11-2	119	95	24	1,48	Y
2Ha 11-3	95	71	24	0	Y
2Ha 11-4	239	182	57	0,18	Y
2Ha 11-5	6	5	1	0,06	Y
2Ha 11-6	30	22	8	0,01	Y
2Ha 13-51	291	-	291	-	N
2Ha 13-52	47	-	47	-	N

(p≤0.05).

Y: Yes.

N: No.

The results from qPCR showed that the GM event 2Ha11 presented from one to four copies of the transgene, randomly inserted in the soybean genome.

### Analysis of gene expression by RT-qPCR

**Figure 1:**
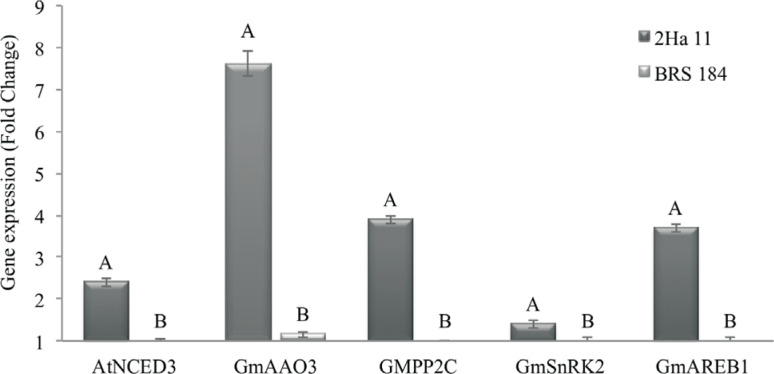
Quantification of relative expression of transgene *AtNCED3* and endogenous genes *GmAREB1* (Glyma.04G039300; Glyma07.G213100; Glyma.02G131700), *GmPP2C* (Glyma.14G195200), *GmSnRK2* (Glyma.02G135500) and *GmAAO3* (Glyma.14G045100) in the GM event 2Ha11 and conventional cultivar BRS 184 (genetic background). Reactions were carried out in three biological and three technical replicates. Bars represents standard error.

The higher expression levels were detected for all analyzed genes in the event 2Ha11 when compared to WT-plants, in WD conditions. The transgene *AtNCED3* presented 2.4 times higher expression in the 2Ha11 event when compared to its genetic background BRS 184, which showed no expression to the transgene. The endogenous genes *GmAREB1* (Glyma.04G039300; Glyma.07G213100; Glyma.02G131700), *GmPP2C* (Glyma.14G195200), *GmSnRK2* (Glyma.02G135500), and *GmAAO3* (Glyma.14gG045100) showed respectively 3.7, 3.9, 1.4, and 7.4 times higher expression in the GM event when compared to BRS 184 plants (Figure 1).

### Growth, physiological and agronomical traits under greenhouse and field conditions for two crop seasons

**Figure 2:**
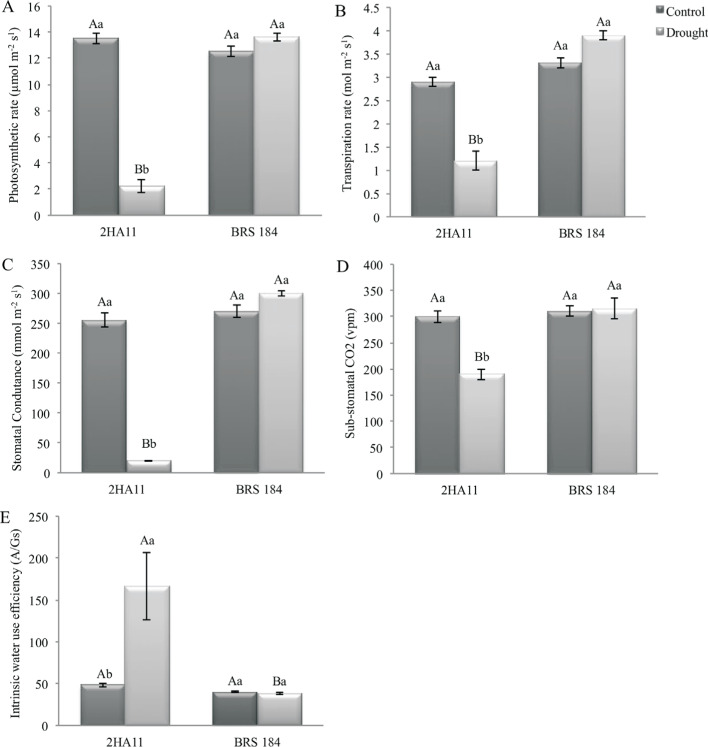
Gas exchange measurements and intrinsic water use efficiency in the conventional soybean cultivar BRS 184 and its isoline, the transgene 2Ha11 *AtNCED3*, under water deficit induced in greenhouse conditions. Means ± standard error of nine replicates. The means followed by the same letters do not differ by the Duncan test (*p*≤0.05). The uppercase letters compare GM line and conventional cultivar (2Ha11/BRS184) and the lowercase letters compare water conditions (control/drought).

The gas exchange measurements (*g_s_, Ci*, *A*, *E*) evaluated in the greenhouse did not show statistical differences between the GM event 2Ha11 and WT plants, under irrigated conditions. However, under WD, these parameters decreased in the event 2Ha11 (Figure 2A-D). Furthermore, the transgenic plants showed 80% higher intrinsic water use efficiency (*A/g_s_*) when compared to WT plants, under WD (Figure 2E). No differences were observed for number of nodes (NN), shoot dry matter, root dry matter, mean length of internodes and relative growth rate in height.

In field conditions, no significant interaction between plant materials and water conditions was observed; probably due to the great amount of rainfall recorded during both crop seasons. The results from field experiments (crop seasons 2015/16 and 2016/17) presented in figures comprise average values from both water conditions (irrigated and non-irrigated treatments), for each cultivar/GM line. The data collected at the weather station showed that the total rainfall recorded was 1,521.4 mm (Figure S2A) and 1,147.2 mm (Figure S2B), respectively, in the 2015/16 and 2016/17 crop-seasons. These volumes were higher than the recommendations for soybean crop, which ranges from 450 to 800 mm/cycle, depending on crop management, general weather conditions and cycle duration (Embrapa, 2014).

**Figure 3:**
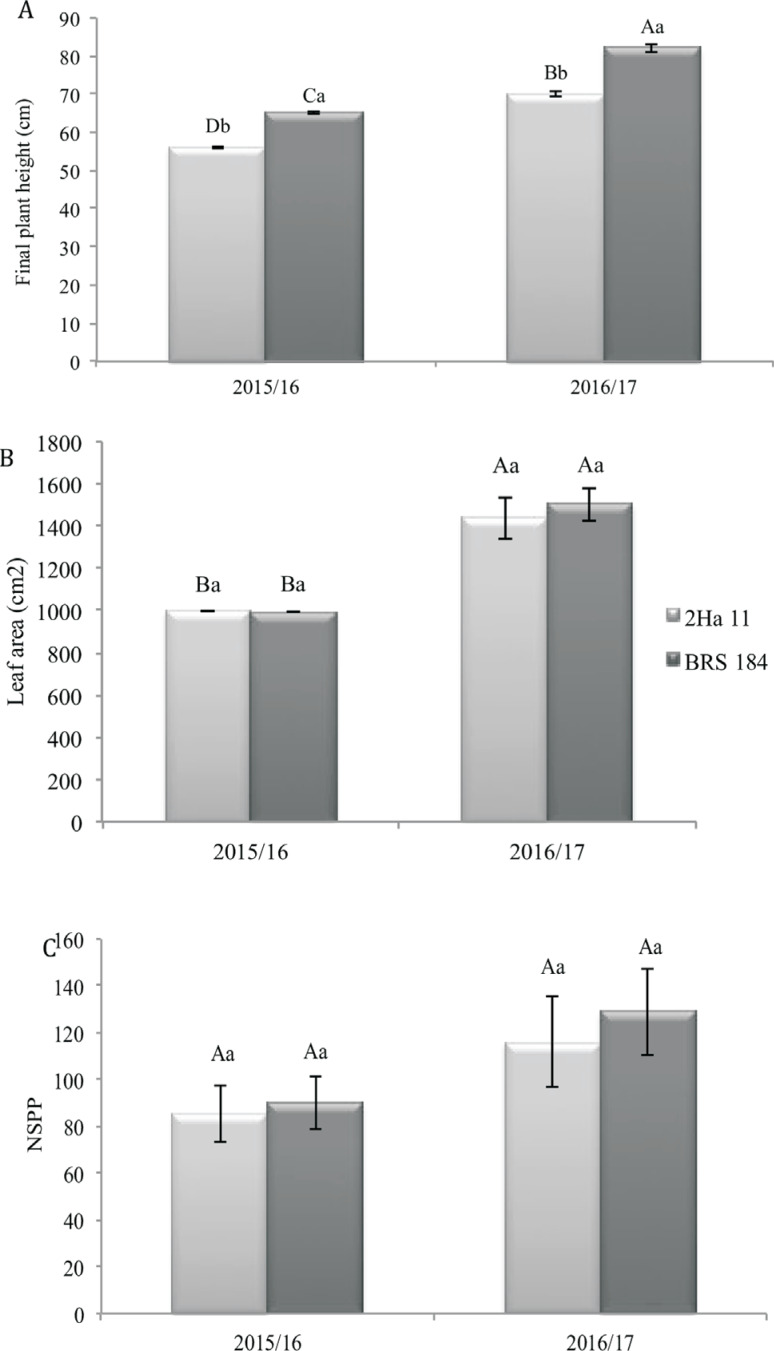
Pllant height, leaf area and number of seeds per plant (NSPP) in the conventional soybean cultivar BRS 184 and its isoline, the transgene 2Ha11 *AtNCED3*, under water deficit induced in field conditions for two crop seasons. Means ± standard error of six replicates. The means followed by the same letters do not differ by the Duncan test (*p*≤0.05). The uppercase letters compare crop seasons (2015/16 and 2016/17) and the lowercase letters compare GM line and conventional cultivar (2Ha11/BRS184).

In greenhouse and field conditions, the 2Ha11 GM line presented similar responses for height, showing on average 12 cm less in greenhouse (data not shown) and 8-9 cm less in field experiments when compared to WT plants (Figure 3A).

**Figure 4:**
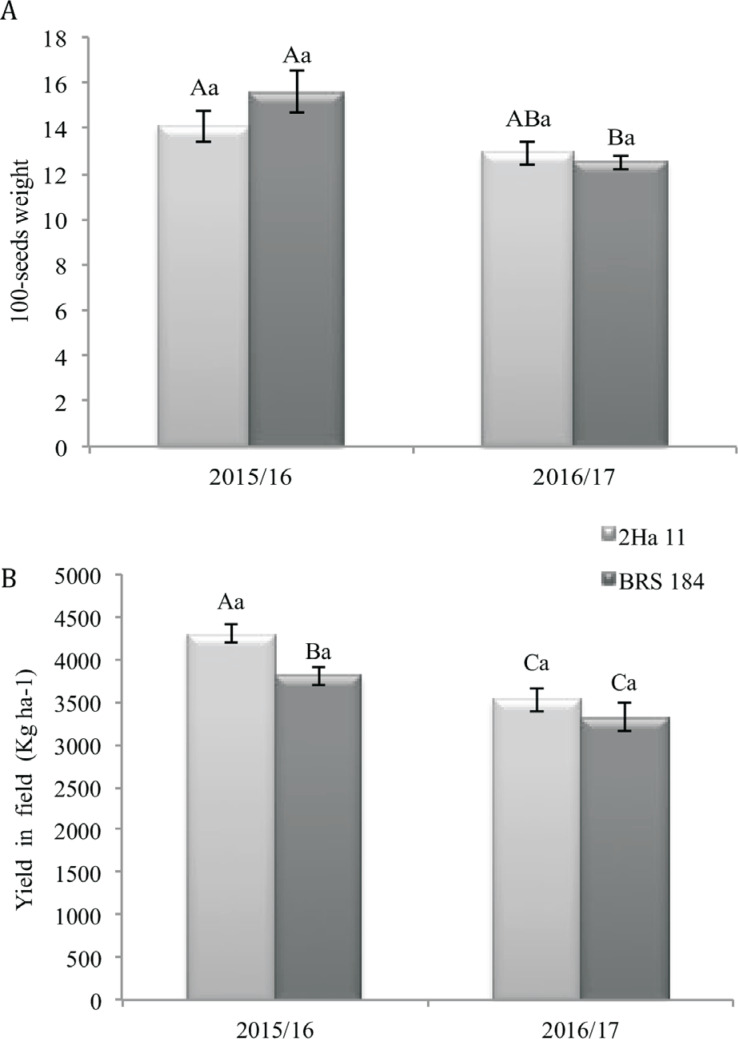
Data from 100-seed weight and yield in the conventional soybean cultivar BRS 184 and its isoline, the transgene 2Ha11 *AtNCED3*, under water deficit induced in field conditions for two crop seasons. Means ± standard error of six replicates. The means followed by the same letters do not differ by the Duncan test (*p*≤0.05). The uppercase letters compare crop seasons (2015/16 and 2016/17) and the lowercase letters compare GM line and conventional cultivar (2Ha11/BRS184).

**Figure 5:**
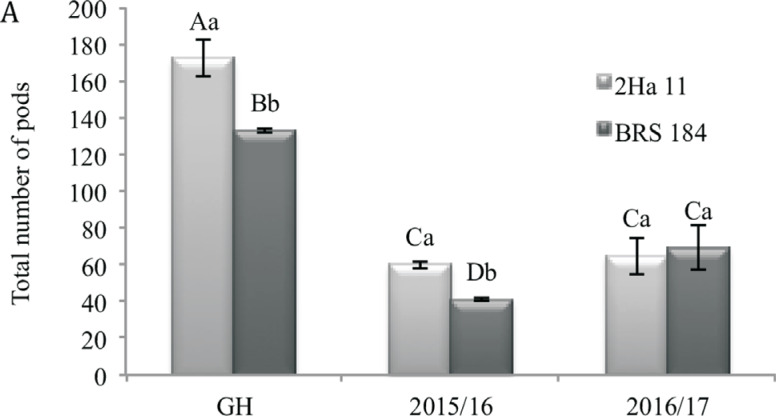
Total number of pods in the conventional soybean cultivar BRS 184 and its isoline, the transgene 2Ha11 *AtNCED3*, under water deficit induced in field conditions for two crop seasons. Means ± standard error of six replicates. The means followed by the same letters do not differ by the Duncan test (*p*≤0.05). The uppercase letters compare results from GH (greenhouse) and crop seasons (2015/16 and 2016/17) and the lowercase letters compare GM line and conventional cultivar (2Ha11/BRS184).

When crop seasons were compared, the GM event 2Ha11 presented lower height (Figure 3A), lower leaf area (Figure 3B), lower number of seeds per plant (Figure 3C), higher 100-seed weight (Figure 4A) although not statically different, and higher yield (Figure 4B) in the crop season 2015/16 than in 2016/17. In the 2015/2016-crop season, the GM event 2Ha11 also showed higher total number of pods (Figure 5A) and yield when compared to the WT plants (Figure 4B).

### ABA quantification in soybean plants submitted to WD in greenhouse

The ABA quantification was performed in GM and WT plants under control and WD (10 days of withholding irrigation) conditions. Obtained data were analyzed statistically by Tukey test (*p*≤0.05). Plants from the event 2Ha11 under WD showed an ABA concentration of 166.34 pmol mL^-1^, while BRS 184 plants under WD and both plant materials under control conditions showed ABA concentrations under 4 pmol mL^-1^, which is the minimum limit detected by the kit.

## Discussion

Among the climatic problems that agriculture has currently faced, drought has mostly affected yield and the economy of producing regions. The solutions to cope with and minimize this environmental stress are urgently needed to maintain suitable levels of productivity and to feed the worldwide growing and starving population in the next decades. Therefore, GM soybean plants containing the *AtNCED3* gene were obtained. The *NCED3* gene encodes for a vital enzyme in the ABA biosynthesis pathway. Briefly, the zeaXanthin epoxidase (ZEP, EC: 1.14.13.90, Zea-epoxidase) (Marin *et al.*, 1996) catalyzes the epoxidation of zeaXanthin to produce epoxycarotenoid; 9-cis-epoxycarotenoid dioxygenase (NCED) (Schwartz *et al.*, 1997). The NCED enzyme catalyzes the cleavage reaction of epoxycarotenoid to produce xanthoxin (the first C15 intermediate); and abscisic aldehyde oxidase (AAO, EC: 1.2.3.14) (Seo *et al.*, 2000), which catalyzes the final step, converting ABA aldehyde to ABA. The xanthophyll cleavage by NCED is rate limiting and the first committed stage in ABA biosynthesis (Nambara and Marion-Poll, 2005).

In general, genetic transformation through *A. tumefaciens* methodology inserts a low number of transgene copies into host genome, reflecting in a Mendelian segregation in T2 generation (Oltmanns *et al.*, 2010), as identified for the event 2Ha11. However, the GM event 2Ha13, obtained independently did not present Mendelian segregation, suggesting a high number of transgene copies inserted. The GMs lines obtained through this methodology are independently events and usually behave differentially, once the transgene is randomized inserted into the genome which can confer instability to the transgene (Dominguez *et al.*, 2002), not being transmitted to further generations as occurred with 2Ha13 line.

Although soybean is considered as recalcitrant for plant transformation, our research group had already reported soybean GM events presenting a low copy number of transgenes (as 2Ha11 line) regardless of the construct (Marinho *et al.*, 2015; Honna *et al.*, 2016). This characteristic is desired when GM lines are incorporated into breeding programs, as it implies in an easier fixation by self-pollination and facilitates the transgene identification in further crosses to develop cultivars more tolerant to drought. Likewise, the expression of the transgene is mandatory. Thus, in the present study, the GM event 2Ha11 showed higher expression levels (6 times more than control condition) of *AtNCED3* and the endogenous genes *GmAREB1*, *GmPP2C*, *GmSnRK2*, and *GmAAO3* under WD (Figure 1), suggesting that *NCED* gene is involved in drought response in soybeans. This expression level was also reported for *Arabidopsis* (Chan, 2012). According to this author, the levels of *NCED* gene can range from −3 to 3 (respectively, down- and up-regulation) in drought condition, similar to what was obtained in soybean, and vary with the time of induction of the stress, being enough to induce drought-responses in the plants. Furthermore, the ABA synthesis pathway was triggered in response to WD in GM event 2Ha11, as observed by higher ABA levels detected under such a condition in the GM plants.

As reported here, in *Arabidopsis*, the overexpression of *AtNCED3* also directed an increase in ABA endogenous levels and induced ABA-genes. Furthermore, *Arabidopsis* plants overexpressing *AtNCED3* presented an improvement in drought tolerance (Iuchi *et al.*, 2001). The participation of *NCED* genes in drought tolerance was also reported in other plant species. Under WD, plants of *Caragana korshinskii*, displayed ABA accumulation in leaves and stems, followed by a significant increase in *CkNCED1* mRNA levels (Wang *et al.*, 2009). Furthermore, in *Stylosanthes guianensis*, *SgNCED1* gene expression was induced in leaves and roots submitted to drought-conditions. Dehydration also strongly and rapidly induced the expression of *SgNCED1,* and the ABA accumulation was coincidently detected with an increase in *SgNCED1* mRNA levels under stress (Yang and Guo, 2007). Moreover, tobacco overexpressing the *SgNCED1* gene showed enhanced drought tolerance, with induced-expression of drought-responsive genes (Bao *et al.*, 2016). These responses were also described to important economic crops, which in some cases was accompanied by a significant increase in the ABA levels, as observed here for soybean. In dehydrated leaves of saffron (*Crocus sativus*), increased levels of *CstNCED* mRNA were detected 2h after detachment, reaching its highest level after 5h (Ahrazem *et al.*, 2012). In avocado (*Persea americana* Mill. cv Lula), *PaNCED1* gene expression was significantly increased by 80% in dehydrated leaves (Chernys and Zeevaart, 2000). In common beans (*Phaseolous vulgaris*), preceding the accumulation of ABA, mRNA and protein levels of *PvNCED1* gene were strongly induced in response to water stress (Quin and Zeevaart, 1999). Furthermore, plants of transgenic tobacco (*Nicotiana plumbaginifolia* Viv.) overexpressing *PvNCED1* gene showed higher ABA levels (Marin and Quesada, 1996). Moreover, cowpea (*Vigna unguiculata*) plants showed accumulation of ABA and expression of *VuNCED1* was strongly induced by water deficit. As reported here in soybean, a high ABA concentration under WD was also identified in *Arabidopsis* plants 10h-dehydrated, which showed 140 times higher ABA levels than un-stressed plants (Iuchi *et al.*, 2000).

Furthermore, in leaves and stems of peanut (*Arachis hypogaea* L.), higher levels of ABA were also reported after 15 days of withholding irrigation, as well as a significant up-regulation of *AhNCED1* under dehydration conditions (Wan and Li, 2006). In citrus (*Citrus sinensis* L. Osbeck), *CsNCED1* gene expression increased in water-deficit treated leaves, in a consistent pattern with ABA accumulation (Rodrigo *et al.*, 2006). Also, in Clemenules mandarin (*Citrus clementina*), during the water scarcity/re-watering rounds, *CcNCED3* gene expression paralleled the pattern of ABA accumulation in leaves (Agustí *et al.*, 2007). In ‘Rangpur’ lime, the *NCED5* gene was highly induced in leaves. Furthermore, in ‘Sunki Maravilha’, *NCED2* gene was also highly expressed in leaves. In both genotypes, the transcription levels of these genes correlated with ABA accumulation in severe WD conditions (Marin *et al.*, 1996). In tomato, an increase in *LeNCED* mRNA levels was reported during drought conditions (Thompson *et al.*, 2000). In soybean, *GmNCED3, GmNCED4* and *GmNCED5* genes higher expression was also reported and showed to be limited by light under treatment conditions, the period in which stomatal closure is required to prevent water loss by evapotranspiration (Rodrigues *et al.*, 2015). As observed in the present study, all these reports corroborate the strong and direct relationship among the expression of *NCED* genes, increased ABA levels and the activation of drought responses, such as stomatal closure, to reduce water loss under WD.

Likewise, as reported in this work for soybean, higher expression of the drought-responsive endogenous genes *GmAREB1*, *GmPP2C*, *GmSnRK2*, and *GmAAO3* was also reported in *Arabidopsis* genetically modified with *AtNCED3* (Iuchi *et al.*, 2001). Considering genes from the ABA pathway, an increase in aldehyde oxidase genes under WD was reported in *Arabidopsis* (Koiwai *et al.*, 2004), pea (Zdunek-Zastocka and Sobczak, 2013) and peanut (Yang *et al.*, 2011). In *A. thaliana*, *AAO3* transcripts presented a strong increase in response to water deficit (Koiwai *et al.*, 2004). In *Arachis hypogaea* L., a dominant expression of the *AhAO2* gene was observed in leaves. The overexpression of *AhAO2* in *Arabidopsis* headed to an increase in ABA levels and improved drought tolerance (Yang *et al.*, 2011). In pea (*Pisum sativum*), during a progressively induced drought treatment, levels of *PsAO3* transcripts increased expressively in roots and leaves, while ABA accumulation was identified only in leaves, complemented by the induction of the *PsNCED3* gene expression (Zdunek-Zastocka and Sobczak, 2013).

The high expression of *GmPP2C* and *GmSnRK2* genes identified in the present study reflects the refined control of ABA synthesis, which is negatively regulated when the ABA levels exceeded, by the inhibition of NCED enzyme, preventing that high hormone levels implies in plant metabolic disturbances (Liu *et al.*, 2016). The initial events in the ABA signaling pathway strike through a central signaling module comprising three classes of proteins: the ABA receptors - PYR/RCARs; the negative regulators - protein phosphatase 2Cs (PP2Cs); and the positive regulators of downstream signaling - SNF1-related protein kinase 2s (SnRK2s) (Ma *et al.*, 2009; Park *et al.*, 2009). A double-negative regulatory pathway is established, whereby ABA-bound PYR/RCARs inhibit PP2C activity and PP2Cs inactivate SnRK2s (Park *et al.*, 2009; Umezawa *et al.*, 2009; Vlad *et al.*, 2009). Therefore, in ABA absence, PP2Cs are active and repress SnRK2 activity. In ABA presence, PYR/RCARs interact with PP2Cs and inhibit phosphatase activity, leading to SnRK2 activation and phosphorylation of target proteins (Hubbard *et al.*, 2010). In land plants, ABA receptors SnRK2, group A PP2Cs, and RCAR/PYR/PYL control ABA signaling pathway including TFs AREB/ABFs Umezawa, Nakashima and Miyakawa, 2010; Nakashima and Yamaguchi-Shinozaki, 2013 and Miyakawa *et al.*, 2013). Thus, SnRK2s phosphorylate ABFs (ABRE-binding factors), conserved ABA-responsive cis-elements (ABRE) present in the promoter region of genes ABA-regulated (Furihata *et al.*, 2006), such as ZIP TFs, AREB1/ABF2, AREB2/ABF4 and ABF3 (Yoshida *et al.*, 2014). Therefore, in soybean, *GmSnRK2* might have activated the expression of *GmAREB1* by phosphorylation.

Data from physiological characterization of *AtNCED3* soybean lines showed that the level of transgene expression detected was able to induce endogenous ABA levels, resulting in plants with more water use efficiency and therefore, performing better not only under greenhouse experiments but also under field conditions. According to the available literature, plants facing low to moderate water deficit will frequently enhance WUE (Brock and Galen, 2005; Liu *et al.*, 2005; Yin *et al.*, 2005; Medrano *et al.*, 2015), maybe as a protective mechanism against stress, by allowing plants to save water and improve its use efficiency (Chaves *et al.*, 2009), mainly with the objective to convert available CO_2_ into photoassimilates in pods and in grain production, and thus reflecting in increased in yield.

The decrease in gas exchange parameters observed in the soybean GM event 2Ha11 under WD has also been described in other plants species. *Arabidopsis* overexpressing *AtNCED3* gene (Iuchi *et al.*, 2001) and *OsNCED3* gene from rice (Hwang *et al.*, 2010) showed a decrease in the transpiration rate in leaves. Furthermore, genetically modified tobacco lines overexpressing *SgNCED1* also showed higher ABA levels, a decrease in transpiration rate and lower photo-synthetic rate (24–47%), as a result of lower stomatal conductance (Zhang *et al.*, 2008). Detached leaves from transgenic tobacco overexpressing *PvNCED1* (*Phaseolous vulgaris*) similarly showed lower water loss by transpiration (Qin and Zeevaart, 2002). Enhanced stomatal closure was also observed in transgenic lines of *Vicia fava* expressing the *AtNCED3* gene (Melhorn *et al.*, 2008). This might also have occurred with the GM event 2Ha11 in the present study, since reduced values of gas exchange parameters were observed under WD conditions (Figure 2). ABA has been widely reported as an inducer of stomatal closure with the aim of reduces water loss by transpiration. This was probably the mechanism activated in all these plants to enhance drought tolerance, i.e. a decrease in stomatal aperture triggered by an increase of the ABA levels.

As observed for the GM event 2Ha11, an increase in yield components was reported in GM lines of creeping bent grass overexpressing *VuNCED1,* which showed increased plant body biomass with increased number of tillers under water deficit conditions (Aswath *et al.*, 2005). Final yield is composed by three parameters, which are number of pods per unit area, number of seeds per pod and average grain weight. Although these components were not presented individually, the higher final yield of GM 2Ha11 can be explained by the combination of these three components. For grain crops, a successful selection approach would eventually be determined by the reproductive achievement and by the final yield (Saint *et al.*, 2012). Therefore, it is very important to trial NCED plants in field conditions, as few studies have described results from GM crops cultivated in genuine field conditions (Passioura, 2012; Saint *et al.*, 2012). In the present study, in a field-experiment, in crop season 2015/16, in relation to its background, the GM event 2Ha11 showed increased yield (Figure 4B), which was probably a result of higher 100-seeds weight (Figure 4A) and total number of pods (Figure 5). Some authors (Rolla *et al.*, 2013; Fuganti-Pagliarini *et al.*, 2017) have also described experiments for soybean GM lines under field conditions in four different water regimes; irrigation, drought (rain-fed) and water deficit treatment induced through rainout shelters at vegetative or reproductive stages. As reported here, the GM plants showed increased yield components when drought was induced. It is also important to highlight that Molinari *et al.* (2018) reported no differences among the *NCED* GM lines and background cultivar in seedling, also an important phase that can be impaired by water deficit and results in low final yield. Yet, no phenotype differences were identified in the GM plants submitted to drought-experiments under greenhouse and field conditions.

The results obtained here from molecular and physiological studies in greenhouse and field conditions confirm the potential of overexpressing *NCED* gene to improve drought tolerance in soybean and suggest that the *AtNCED3* soybean plants might outperform under drought, reducing economic and yield losses, thus being a good candidate line to be incorporated in the soybean-breeding program to develop drought-tolerant cultivars.

## Supplementary material

The following online material is available for this article:

Figure S1 Plasmid pC3300J-35S-AtNCED3.

Figure S2 Water balance from crop seasons 2015-2016 and 2016-2017.

Table S1 Chi-square (X^2^) test for Mendelian segregation pattern.
