# Supplementary material for: Overexpression of AtNCED3 gene improved drought tolerance in soybean in greenhouse and field conditions
Source: Genet Mol Biol. 2020 Jun 8;43(3):e20190292. doi: 10.1590/1678-4685-GMB-2019-0292 (PMC7278712; doi:10.1590/1678-4685-GMB-2019-0292)
Supplement: Supplementary file 1 [file 1415-4757-GMB-43-3-e20190292-suppl1.pdf]

Genetics and Molecular Biology
https://doi.org/10.1590/1678-4685-GMB-2019-0292

Supplementary Material to "Overexpression of AtNCED3 gene improved drought tolerance in soybean in greenhouse and field conditions"

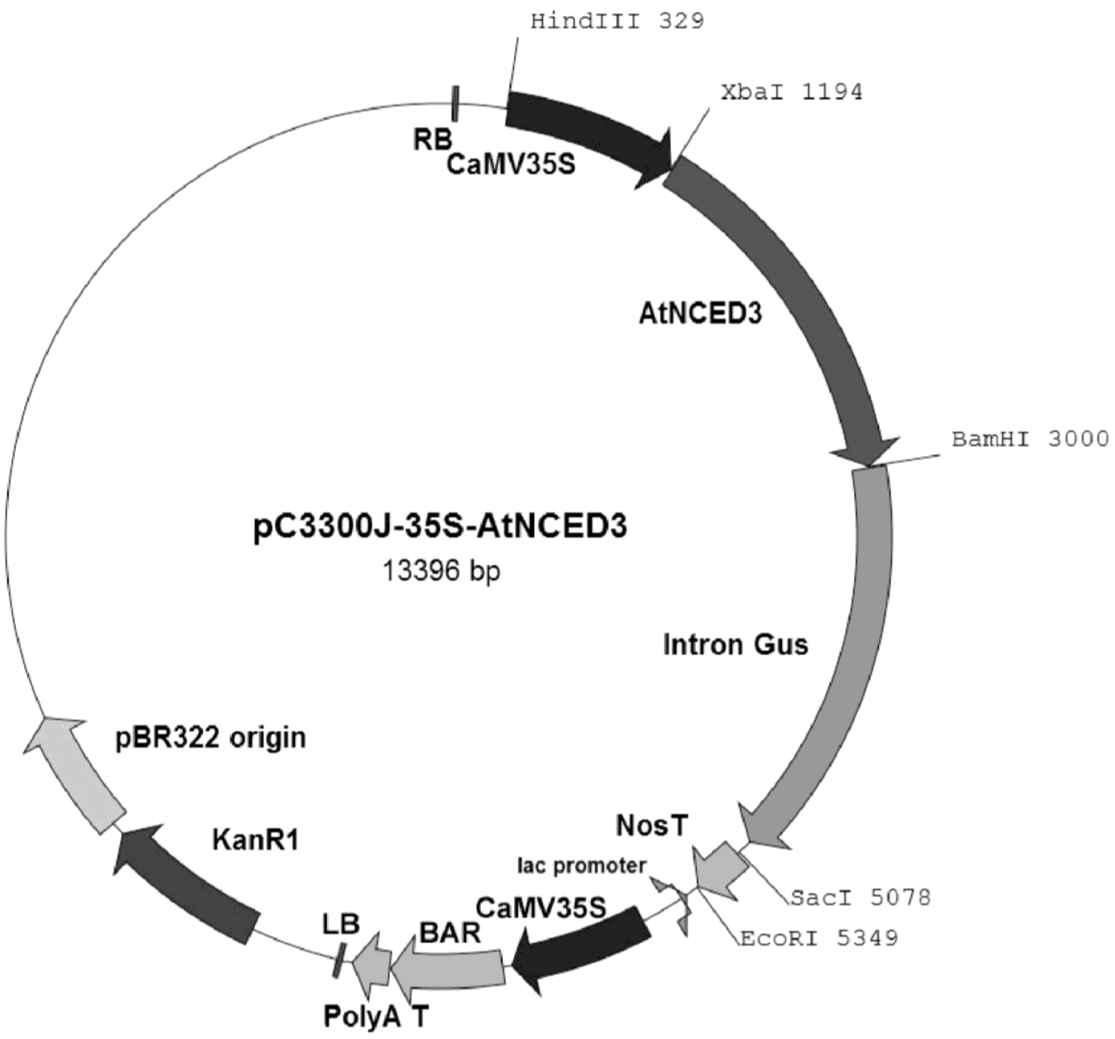

Figure S1 - Plasmid pC3300J-35S-AtNCED3. This vector is under the control of the constitutive promoter CaMV 35S (Cauliflower mosaic virus) and TNOS terminator (*A. tumefaciens* nopaline synthase). Two marker genes are also present in the cassette structure: the *bar* gene (phosphinothricin acetyl transferase), which confers resistance to the herbicide ammonium glufosinate, used as a selective agent; and the *NPTII* gene (Neomycin phosphotransferase), which confers resistance to the antibiotic kanamycin, used to select the colonies containing the inserted transgene.
