# Supplementary material for: Overexpression of AtNCED3 gene improved drought tolerance in soybean in greenhouse and field conditions
Source: Genet Mol Biol. 2020 Jun 8;43(3):e20190292. doi: 10.1590/1678-4685-GMB-2019-0292 (PMC7278712; doi:10.1590/1678-4685-GMB-2019-0292)
Supplement: Supplementary file 2 [file 1415-4757-GMB-43-3-e20190292-suppl3.pdf]

Genetics and Molecular Biology
https://doi.org/10.1590/1678-4685-GMB-2019-0292

Supplementary Material to "Overexpression of AtNCED3 gene improved drought tolerance in soybean in greenhouse and field conditions"

Table S1 - In A, primer sequences used to identity positive events for *AtNCED3* construct by conventional PCR; in B, Primer sequences for *AtNCED3* and *GmLectina* genes, used to perform gene expression quantification by RT-qPCR and in C, primer sequences for *AtNCED3, GmAAO3, GmAREB1, GmSnRK2, GmPP2C* and *Gmβ-actina* genes used to perform gene expression quantification by RT-qPCR.

A Primer sequences used to identity positive events for *AtNCED3* construct by conventional PCR.

Primers	Sequences (5'-3')	Amplicon size (bp)
AtNCED3-F	TCTAGAATGGCTTCTTTCACGGCAAC	979
AtNCED3-R	GGATCCTCACACGCACTGCTTCGCCA	
GmGUS-F	CGTCCTGTAGAAACCCC	1813
GmGUS-R	GCGCGGTACGGTAGGAGTTG	

B. Primer sequences for *AtNCED3* and *GmLectina* genes, used to perform gene expression quantification by RT-qPCR.

Primers	Sequences (5'-3')	Amplicon size (bp)
AtNCED3-F	CAAGTTCAGATCACTCCCAA	129
AtNCED3-R	GCTTAAAGCGAAGAGTTCAC	
GmLectina-F	TCCCGAGTGGGTGAGGATAG	85
GmLectina-R	CATGCGATTCCCCAGGTATG	

C. Primer sequences for *AtNCED3*, *GmAAO3*, *GmAREB1*, *GmSnRK2, GmPP2C* and *Gmβ-actina* genes used to perform gene expression quantification by RT-qPCR.

Primers	Sequences (5' – 3')	Amplicon size (bp)
AtNCED3-F	CAAGTTCAGATCACTCCCAA	129
AtNCED3-R	GCTTAAAGCGAAGAGTTCAC	
GmAAO3-F	ACACTACTTTGCAGCATACA	142
GmAAO3-R	CACACATTCCAGGAGTACAA	
GmAREB1-F	AATAGAGAATCAGCTGCGAG	80
GmAREB1-R	GCTGAGGTTGCAAAACTTAA	
GmPP2C-F	GCTATGTTGATTTATGCCGTGGTG	115
GmPP2C-R	ACTTTGGTCTCAGGCTCTGCTGTCA	
GmSnRK2-F	CAAAGTGATCTCATGGATGGGA	215
GmSnRK2-R	TGCTATCTAAGTCAAGGTCAGGATC	
Gmβ-actina-F	GAGCTATGAATTGCCTGATGG	118
Gmβ-actina-R	CGTTTCATGAATTCCAGTAGC	
