# Supplementary material for: Overexpression of AtNCED3 gene improved drought tolerance in soybean in greenhouse and field conditions
Source: Genet Mol Biol. 2020 Jun 8;43(3):e20190292. doi: 10.1590/1678-4685-GMB-2019-0292 (PMC7278712; doi:10.1590/1678-4685-GMB-2019-0292)
Supplement: Supplementary file 3 [file 1415-4757-GMB-43-3-e20190292-suppl2.pdf]

Genetics and Molecular Biology
https://doi.org/10.1590/1678-4685-GMB-2019-0292

Supplementary Material to "Overexpression of AtNCED3 gene improved drought tolerance in soybean in greenhouse and field conditions"

A

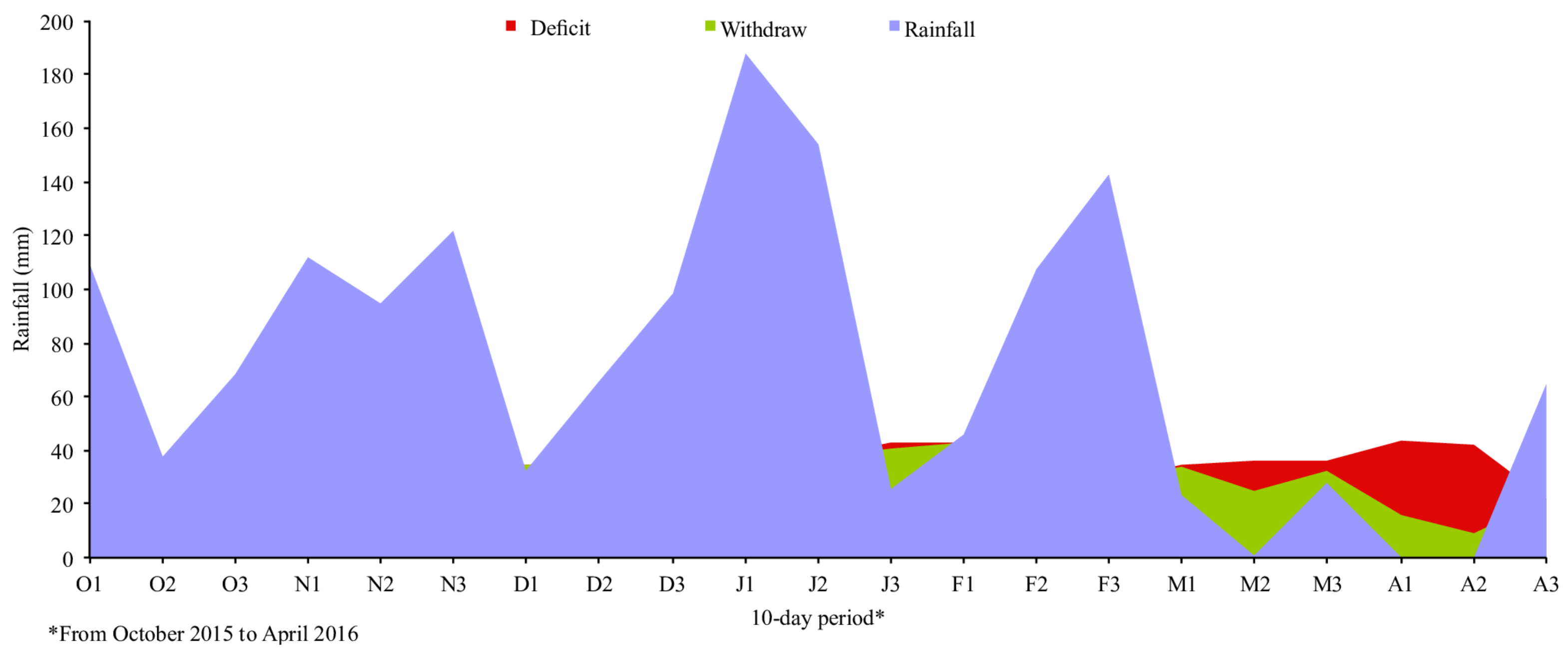

B

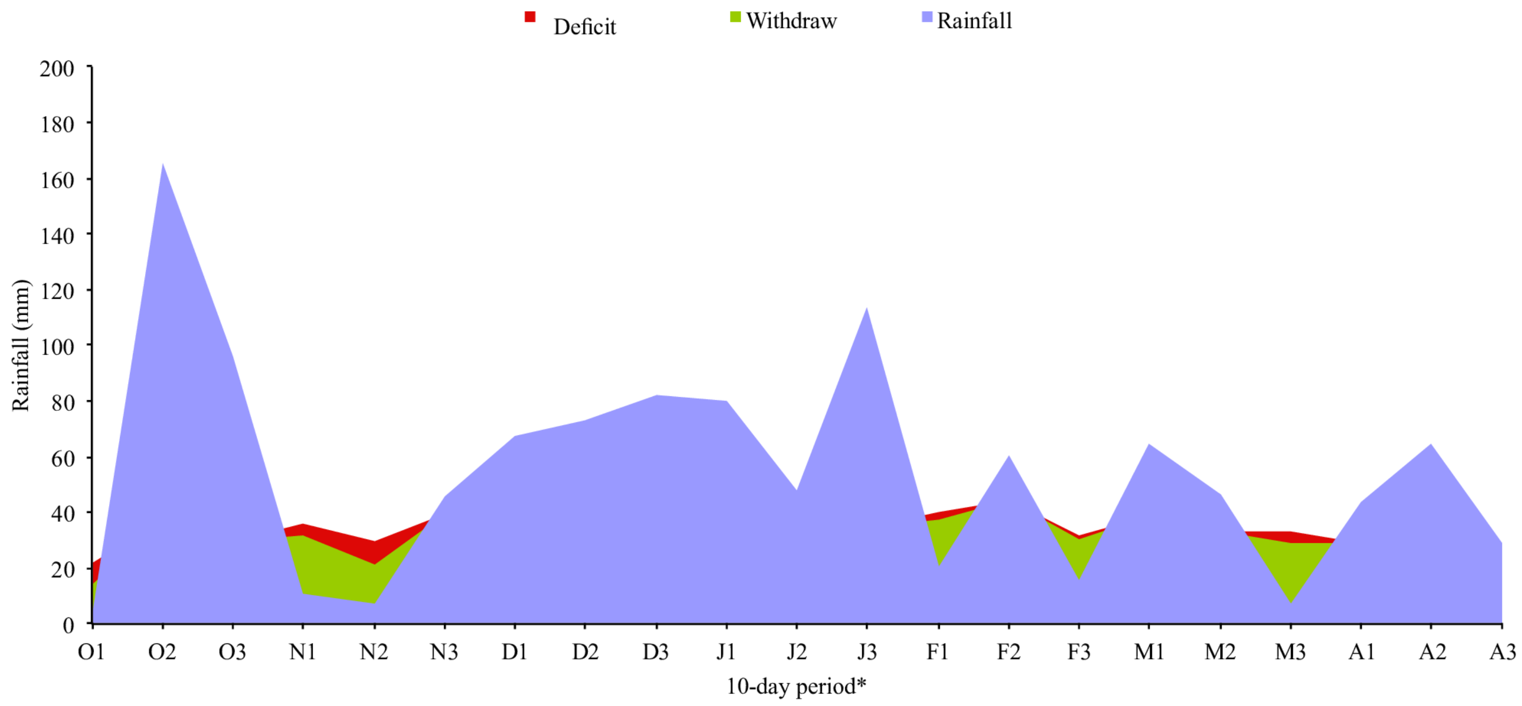

* From October 2016 to April 2017

Figure S2 - In A, water balance from crop seasons 2015-2016 and in B, from 2016-2017. Data were obtained from October to April, every 10-days period. Graphics show rainfall, deficit and water withdraw.
